# Photocatalytic Nanofabrication and Intracellular Raman Imaging of Living Cells with Functionalized AFM Probes

**DOI:** 10.3390/mi11050495

**Published:** 2020-05-13

**Authors:** Takayuki Shibata, Hiromi Furukawa, Yasuharu Ito, Masahiro Nagahama, Terutake Hayashi, Miho Ishii-Teshima, Moeto Nagai

**Affiliations:** 1 Department of Mechanical Engineering, Toyohashi University of Technology, Toyohashi, Aichi 441-8580, Japan; h-furukawa@mems.me.tut.ac.jp (H.F.); y-itou@mems.me.tut.ac.jp (Y.I.); m-nagahama@mems.me.tut.ac.jp (M.N.); teshima@me.tut.ac.jp (M.I.-T.); nagai@me.tut.ac.jp (M.N.); 2 Department of Mechanical Engineering, Kyushu University, Fukuoka 819-0395, Japan; thayashi@mech.kyushu-u.ac.jp

**Keywords:** atomic force microscopy (AFM), functionalized AFM probes, TiO_2_-mediated photocatalytic nanofabrication, tip-enhanced Raman spectroscopy (TERS), intracellular TERS imaging

## Abstract

Atomic force microscopy (AFM) is an effective platform for in vitro manipulation and analysis of living cells in medical and biological sciences. To introduce additional new features and functionalities into a conventional AFM system, we investigated the photocatalytic nanofabrication and intracellular Raman imaging of living cells by employing functionalized AFM probes. Herein, we investigated the effect of indentation speed on the cell membrane perforation of living HeLa cells based on highly localized photochemical oxidation with a catalytic titanium dioxide (TiO_2_)-functionalized AFM probe. On the basis of force–distance curves obtained during the indentation process, the probability of cell membrane perforation, penetration force, and cell viability was determined quantitatively. Moreover, we explored the possibility of intracellular tip-enhanced Raman spectroscopy (TERS) imaging of molecular dynamics in living cells via an AFM probe functionalized with silver nanoparticles in a homemade Raman system integrated with an inverted microscope. We successfully demonstrated that the intracellular TERS imaging has the potential to visualize distinctly different features in Raman spectra between the nucleus and the cytoplasm of a single living cell and to analyze the dynamic behavior of biomolecules inside a living cell.

## 1. Introduction

Recent progress in single-cell manipulation and analysis, particularly on microfluidic platforms, has opened up new possibilities in a wide range of research areas, from basic biology to biomedical applications such as medical diagnostics, drug discovery, and tissue engineering [1,2,3]. This approach is crucial to enable a comprehensive understanding of life processes at the cellular level, thus further improving human health and quality of life. Atomic force microscopy (AFM) also provides an effective platform for in vitro manipulation and analysis of single living cells in biological sciences and medicine, owing to its unique capabilities with high spatial and temporal resolution [4,5,6,7,8]. It is capable of allowing topographical imaging and morphological characterization of native biological samples from DNA and proteins to living cells at the molecular level under near-physiological conditions. Further, it can characterize the mechanical properties of these cells, such as elasticity and viscoelasticity, and enable the quantitative mapping of their localized properties with a nanoscale spatial resolution by scanning the whole cell surface [9,10,11,12,13]. It has also opened up a new area of research on adaptation processes in living cells in response to mechanical stimuli, which is crucial for various cellular processes including cell growth, proliferation, differentiation, and even cell death [14,15,16,17]. This emerging field is currently referred to as cellular mechanobiology, which can be considered as three successive steps comprising mechanosensation, mechanotransduction, and mechanoresponse. AFM-based force spectroscopy provides an effective platform for in vitro manipulation of single biomolecules and cells, thus enabling a direct measurement of interaction forces between biomolecular pairs (e.g., DNA, RNA, proteins, or drug molecules) and adhesion forces between living cells with piconewton force sensitivity and at the single-molecule level [18,19,20,21,22,23,24]. Additionally, the advent of high-speed AFM has recently allowed us to study dynamic structural changes and dynamic processes of functioning biological molecules at high spatiotemporal resolution [20,24,25,26]. The combination of force spectroscopy with high-speed AFM has provided important insights into single-molecule mechanics, particularly in single protein unfolding and receptor–ligand complexes [27].

Another challenge is how to introduce biomolecules or therapeutic drugs into living cells and extract intracellular biomolecules or components with minimal damage to the cells [28]. This approach is crucial for addressing the fundamental analysis of cellular functions, mechanisms in living systems, genetic transformation, and cell-based assays. Han et al. successfully delivered plasmid DNA containing the green fluorescent protein (GFP) gene into a living cell with high efficiency by inserting an AFM tip sharpened to the shape of an ultrathin needle, on which the DNA was adsorbed [29]. Uehara et al. demonstrated that cytoplasmic components containing mRNA can be extracted from a living cell by inserting a pyramidal AFM tip; in this process, the mRNA is successfully attached to the surface of the tip by physical absorption [30]. However, both techniques based on physical absorption are limited in their ability to introduce and extract desired biomolecules. Two similar alternative approaches have been proposed for enabling intra- and extra-cellular delivery of biomolecules together with a variety of conventional AFM functions by employing newly designed AFM probes consisting of a microchanneled cantilever and a hollow tip with a submicrometer aperture instead of a conventional solid cantilever beam structure and solid sharp tip, to facilitate liquid handling [31,32]. Among them, fluidic force microscopy (FluidFM) is commercially available and provides an innovative platform in molecular and cellular biology [33,34].

Raman spectroscopy is also a powerful analytical tool widely used in almost all research fields of natural sciences and technologies to provide a chemical fingerprint for molecular identification [35,36]. However, spontaneous Raman scattering is typically very weak. Therefore, tip-enhanced Raman spectroscopy (TERS) has emerged as a novel label-free and non-destructive technique for the simultaneous mapping of topographic and chemical information at the nanoscale. It combines the high sensitivity of surface-enhanced Raman spectroscopy (SERS) with the high spatial resolution of AFM, which enhances Raman scattering by molecules adsorbed on the AFM tip functionalized with metallic nanostructures such as gold (Au) or silver (Ag) nanoparticles [36,37,38]. The advent of the TERS allows us to characterize a variety of biological samples, ranging from biomacromolecules such as nucleic acids (DNA and RNA) and proteins to more complex biological systems such as pathogens (bacteria and viruses) and living cells [36,39,40]. Recently, Xiano et al. demonstrated the potential of TERS to investigate a specific interaction between a peptide ligand and a membrane protein receptor on a cancer cell [41]. Additionally, Cowcher et al. demonstrated that TERS can be used to identify differences between the native and glycosylated forms of bovine pancreatic ribonuclease (RNase) [42]. However, only a few attempts have been made to apply TERS in intracellular imaging of biomolecules in living cells. Vitol et al. demonstrated that TERS can be used for in situ analysis of a living cell function in real time by inserting a glass nanopipette tip coated with Au nanoparticles into the cell [43,44].

To introduce novel AFM applications in biological sciences, particularly in cellular function analysis, we have proposed the photocatalytic nanofabrication of living cells under near-physiological conditions based on highly localized photochemical oxidation with a catalytic titanium dioxide (TiO_2_)-functionalized AFM probe under UV irradiation for minimally invasive intracellular delivery. This was described in our previous work [45]. In this study, we further investigated the effect of indentation speed on cell membrane perforation. In the experiments, the probability of the cell membrane perforation and the penetration force were discussed according to force–distance curves obtained during the indentation process. The cell viability was also examined. Moreover, as another functionality of AFM, we have demonstrated intracellular TERS imaging for analyzing the dynamic behavior of biomolecules in a living cell using AFM probes functionalized with silver nanoparticles (AgNP) with a homemade Raman system, which was integrated into an inverted microscope.

## 2. Experimental Methods

### 2.1. Cell Membrane Perforation Using TiO_2_-Functionalized AFM Probes

**Figure 1:**
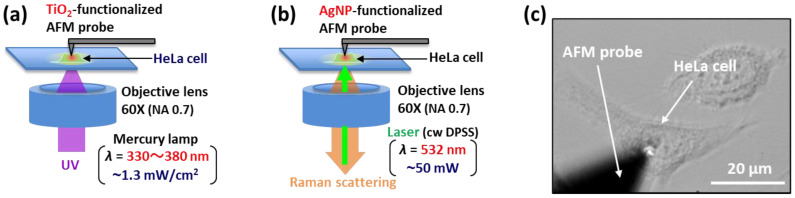
Experimental setups for (**a**) cell membrane perforation based on a highly localized TiO_2_-mediated photocatalytic oxidation, and (**b**) intracellular tip-enhanced Raman spectroscopy (TERS) imaging of molecular dynamics in living cells. (**c**) Optical microscopy image showing an example of insertion tests or intercellular imaging for a living HeLa cell conducted using the atomic force microscopy (AFM) system after the penetration of the cell membrane using a TiO_2_- or AgNP-functionalized AFM probe.

For the AFM-based nanofabrication in living cells [45], TiO_2_-functionalized AFM probes (tip radius: ~50 nm) were prepared by anodic oxidation of titanium thin films (100 nm thickness) sputtered on Si-based AFM probes (AC200TN, Olympus, Tokyo, Japan; nominal spring constant: 9 N/m, nominal tip radius: ~7 nm), which had been previously thermally oxidized up to a thickness of approximately 80 nm. The anodization was performed in a 1 M H_2_SO_4_ electrolyte solution under optimal conditions with a DC voltage of 140 V for 5 min. Under these conditions, single-phase anatase TiO_2_ thin films can be obtained on the surface of the AFM tip [45]. As shown in Figure 1a, TiO_2_-mediated photocatalytic cell membrane perforation was conducted with a commercially available AFM system (Asylum Research MFP-3D-BIO, Oxford Instruments, Abingdon, UK), which was placed on an inverted microscope (Ti-U, Nikon, Tokyo, Japan), using the fabricated TiO_2_-coated AFM probes under UV irradiation (Nikon C-HGFI Intensilight Fiber Illuminator). In the experiments, HeLa cells adhered to the glass bottom dish were used as representative somatic cells (see Figure 1c). Prior to the insertion tests, the culture medium was replaced with phosphate-buffered saline (PBS). UV light (~1.25 mW/cm^2^) in the 330–380 nm in wavelength range, which was selected with an optical filter (Nikon UV-2A), was carefully aligned and focused on the initial contact point between the surface of the cell membrane and the tip apex of the AFM probe with a microscope objective lens (60×, NA = 0.7).

### 2.2. Intracellular TERS Imaging Using AgNP-Functionalized AFM Probes

To add another analytical capability to the AFM, we explored the possibility of intracellular TERS imaging of molecular dynamics in living cells. AgNP-functionalized AFM probes (tip radius: ~50 nm) were obtained by sputtering Ag thin films (thickness: 30 nm) on Si-based AFM probes (Olympus AC200TN), which are the same as those used for TiO_2_-functionalized AFM probes. To perform TERS experiments, we designed and constructed a compact Raman spectroscopy system (250 mm × 350 mm × 350 mm) integrated with an inverted microscope (Nikon Ti-U), which was enclosed inside an acoustic isolation enclosure for AFM measurements (see Figure S1 in the Supplementary Materials). Figure 1b shows the schematics of the experimental setup for intracellular TERS imaging of molecular dynamics in living cells. The excitation light source was a diode-pumped solid-state (DPSS) laser (Samba 532-50, Cobolt AB, Solna, Sweden) with the wavelength of 532 nm and the maximum laser power of 50 mW. After the beam conditions were adjusted, the laser light was focused onto an AFM tip and a sample through a 60× objective lens (NA = 0.7, spot diameter: 1.8 µm). The backscattered Raman signal obtained inside the cell was collected with the same objective and detected by a high-resolution spectrometer (C11713CA, Hamamatsu Photonics, Shizuoka, Japan; spectral resolution: 10 cm^−1^).

## 3. Results and Discussion

### 3.1. Photocatalytic Nanofabrication of Living Cells

#### 3.1.1. Cell Penetration Experiments

**Figure 2:**
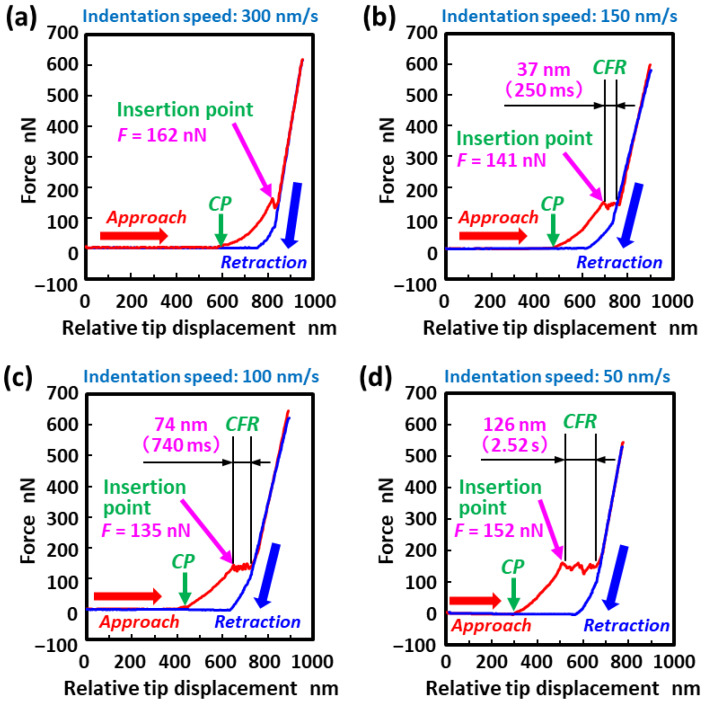
Typical force–distance curves obtained during the insertion of a TiO_2_-functionalized AFM tip into a living HeLa cell under UV irradiation at indentation speeds of (**a**) 300, (**b**) 150, (**c**) 100 and (**d**) 50 nm/s, respectively. The contact points between the AFM tip and the cell surface and the constant force region are denoted by *CP* and an arrow and the symbol *CFR*, respectively.

In our previous work [45], we demonstrated the direct membrane perforation of living cells based on highly localized photochemical oxidation with a catalytic TiO_2_-functionalized AFM probe under UV irradiation. In this study, we further investigated the effect of indentation speed on cell membrane perforation. Figure 2a shows a typical force–distance curve during an insertion of an AFM tip into a living HeLa cell at the indentation speed of 300 nm/s. The contact point between the AFM tip and the cell surface (denoted by *CP* and an arrow in the graphs) was determined as the instant when the force began to increase in the force–distance curves obtained during the approach cycle (red line). When the indentation depth reached approximately 230 nm during the approach cycle, a force drop was observed. This phenomenon indicates the moment when the AFM tip penetrates through the cell membrane [46]. Thereafter, the force increased again, most likely owing to pushing the AFM tip against the surface of the nucleus. This was likely a result of the nucleus being 3–10 times stiffer than the cytoplasm [47]. The resulting penetration force was 162 nN. Figure 2b shows the experimental results when the indentation speed was reduced to 150 nm/s. Similarly, when the indentation force reached 141 nN after the tip of the probe came into contact with the cell membrane, the force temporarily decreased. However, an unusual behavior was observed—the force remained almost constant (see the constant force region denoted by *CFR* in the graphs). As shown in Figure 2c,d, this phenomenon became more pronounced as the indentation speed decreased.

**Figure 3:**
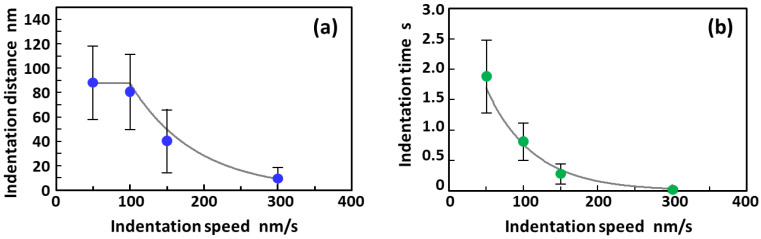
Effects of indentation speed on (**a**) indentation distance and (**b**) indentation time in the constant force region (denoted by *CFR* in Figure 2) when a TiO_2_-functionalized AFM tip was being inserted into a living HeLa cell. Note that each point and error bar in the plot represent the mean values and their standard deviations, respectively.

Figure 3 shows the effects of indentation speed on indentation distance and time, which correspond to the moving distance of the AFM tip downward and the elapsed time while in the constant force region (denoted by *CFR* in Figure 2), respectively. Table 1 presents the detailed numerical data (the mean values and their standard deviations). The probability that the constant force region appeared in the force–distance curves obtained at each indentation speed is also presented in the table. As shown in Figure 3a, the indentation distance during maintaining a constant force increased with a decrease in the indentation speed, but it became constant at speeds of less than 100 nm/s. At an indentation speed of 300 nm/s, the probability that the constant force region appeared in the force–distance curves was 55% (*n* = 20) when cell membrane perforation occurred. However, the indentation distance in the constant force region was estimated to be as small as 10 ± 9 nm, which is equivalent to 40 ± 30 ms in indentation time (see Figure 3b and Table 1). This value for the indentation distance is quite similar to the thickness of the cell membrane (~7 nm) [48].

In contrast, the probability reached 100% (*n* = 7) when the indentation speed was reduced to 150 nm/s (see Table 1). Moreover, the indentation distance and time increased by 40 ± 26 nm and 270 ± 170 ms, respectively. Further decreases in indentation speed by 100 nm/s and 50 nm/s indicate that the indentation distances further increased by 80 ± 31 nm (*n* = 10) and 94 ± 30 nm (*n* = 20), respectively, with a 100% probability of occurrence. These values for the indentation distance are equivalent to 0.80 ± 0.31 s and 1.88 ± 0.61 s in indentation time, respectively. The resulting constant force (in the region *CFR* in Figure 2) indicates that only an extremely small force (less than the force sensitivity) is applied to the AFM probe by the cell membrane despite the spherical (parabolic)-shaped tip of the probe being pressed against it. The results suggest that when the spherical tip apex of the probe is inserted into the cell, the pore diameter created in the cell membrane was widened owing to the photocatalytic degradation of the cell membrane molecules rather than by mechanical force. Additionally, a low probability of occurrence for a relatively high indentation speed of 300 nm/s indicates that sufficient reaction time is required for the decomposition and removal of the cell membrane near the tip of the probe, based on the highly localized TiO_2_-mediated photocatalytic oxidation.

**Figure 4:**
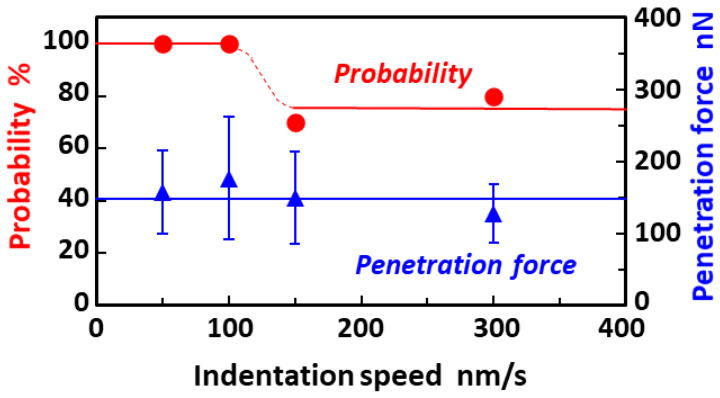
Probability of cell membrane perforation and penetration force obtained with a TiO_2_-functionalized AFM probe under UV irradiation as a function of indentation speed. The vertical bars represent the standard deviation of the penetration force. Note that each point and error bar in the plot of the penetration force represent the mean values and their standard deviations, respectively.

Figure 4 shows the effects of indentation speed on the probability of cell membrane perforation and penetration force obtained with a TiO_2_-functionalized AFM probe under UV irradiation (Table 2 presents the detailed numerical data). Note that the penetration forces are represented by the mean values and their standard deviations. At an indentation speed of 300 nm/s, the probability of cell membrane perforation was estimated to be 80% (*n* = 25). This value even when using the blunt-tipped probe (tip radius: ~50 nm) was nearly twice as large compared with 46% obtained by an uncoated Si-based AFM probe with a sharp tip radius of approximately 7 nm, as described in our previous work [45]. These results suggest that TiO_2_-mediated photocatalytic cell membrane perforation can be successfully achieved. The probability of cell membrane perforation reached 100% at indentation speeds less than 100 nm/s. In contrast, the penetration forces were kept almost constant and were not affected by the indentation speed, according to the fact that there were no statistically significant differences in the penetration forces as the *p*-values obtained in all possible combinations of the forces were greater than 0.05. It should be noted that the resulting penetration forces are much larger than those observed in previous reports [46,49,50], which featured values ranging between 0.5 and 20 nN, which were measured using AFM probes with relatively low spring constants (0.1–1.0 N/m) when compared with a nominal spring constant of 9 N/m used in these experiments. Our experimental data also featured penetration forces of 0.2–0.4 nN, using AFM probes with spring constants of 0.1 N/m (data not shown). This major inconsistency arises owing to stiffness mismatching between the AFM probe and the soft sample (living cell). A probe with greater stiffness will show significantly greater penetration force [49,51]. Another possible reason is that the tip radius (~50 nm) of TiO_2_-functionalized AFM probes is much larger than the tip radii used in previous reports (tip radius: ~10 nm).

#### 3.1.2. Evaluation of Cell Viability

**Figure 5:**
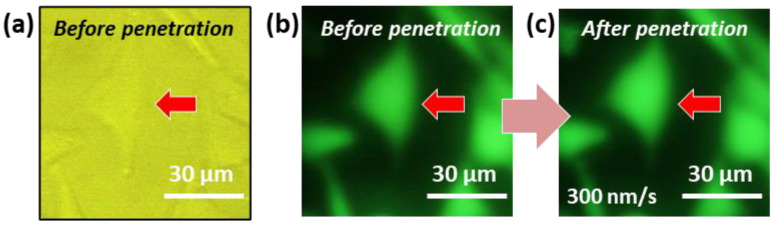
Evaluation of cell viability after cell membrane perforation. (**a**) Bright-field microscopy image and (**b**) fluorescence microscopy image of a living HeLa cell that had been stained with 8 µM Calcein-AM before cell membrane perforation. (**c**) Fluorescence microscopy image of the HeLa cell after the penetration of the cell membrane by a TiO_2_-functionalized AFM probe at an indentation speed of 300 nm/s under UV irradiation.

Cell viability after cell membrane penetration was estimated with Calcein-AM (Dojindo Molecular Technologies, Kumamoto, Japan); this dye stains only living cells, and causes the emission of a strong green fluorescence. As shown in Figure 5, a HeLa cell showed intense green fluorescence even after the cell membrane perforation at an indentation speed of 300 nm/s, indicating that the cell was still alive after insertion of the AFM tip. The cell viability was estimated to be 100%; all the cells (*n* = 8) remained alive after the perforation. Additionally, at an indentation speed of 50 nm/s, cell membrane perforation with a cell viability of 100% (*n* = 10) was also successfully achieved without significant cell damage. Thus, this technique with a high probability of cell viability after cell membrane penetration may enable a minimally invasive delivery of biomolecules, including DNA, RNA, peptides, proteins, and other various small molecules, into almost any type of cell.

### 3.2. Intracellular TERS Imaging of Living Cells

**Figure 6:**
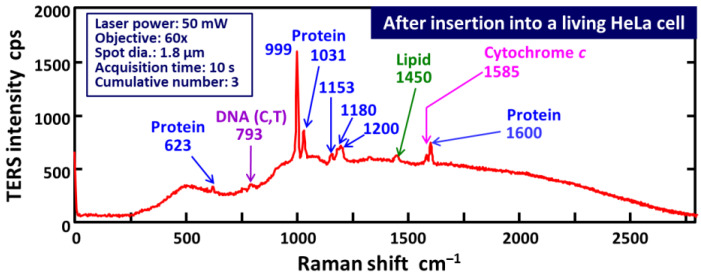
Intracellular TERS spectrum collected by the homemade Raman system after inserting an AgNP-functionalized AFM tip into a living HeLa cell (laser power: 50 mW, acquisition time: 10 s, cumulative number: 3).

Figure 6 shows a typical Raman spectrum (laser power: 50 mW, acquisition time: 10 s, cumulative number: 3) obtained after AgNP-functionalized AFM tip insertion into a living HeLa cell at an indentation speed of 300 nm/s. In all the experiments, the force–distance curves were measured and verified prior to TERS imaging to confirm that the AFM tip had penetrated the cell membrane, which was confirmed by a drop in force appearing in the force curves (see Figure S2 in the Supplementary Materials). As expected, in Figure 6, the AgNP-functionalized AFM tip successfully enhanced inherently weak Raman signals owing to plasmonic field enhancement [36,37,38]; therefore, the peaks most likely associated with DNA (793 cm^−1^), proteins (e.g., 999 cm^−1^), and lipids (1450 cm^−1^) were clearly observed inside the cell [52,53,54,55]. The strong Raman peak at 999 cm^−1^ can be assigned to the breathing mode of phenylalanine [53]. Moreover, several Raman peaks (e.g., 623, 1031, 1153, 1180, 1200, and 1600 cm^−1^) are most likely derived from proteins [54]. The Raman band located at approximately 793 cm^−1^ is primarily attributable to the ring breathing mode of nucleic acid bases (cytosine, thymine, and uracil) and DNA backbone (O–P–O symmetric stretch of the phosphodiester bond in DNA) [54,55]. The peak appearing at 1450 cm^−1^ is likely attributable to the C–H bending modes of phospholipids [52,53]. Moreover, this Raman spectrum includes a very interesting and informative peak; it is Raman scattering from cytochrome *c* (1585 cm^−1^) that triggers the process of apoptosis [56]. It is also noted that the intensities of three representative Raman peaks derived from proteins (at approximately 1000, 1200, and 1600 cm^−1^) increased almost linearly with laser power ranging from 10 to 50 mW (see Figure S3 in the Supplementary Materials).

**Figure 7:**
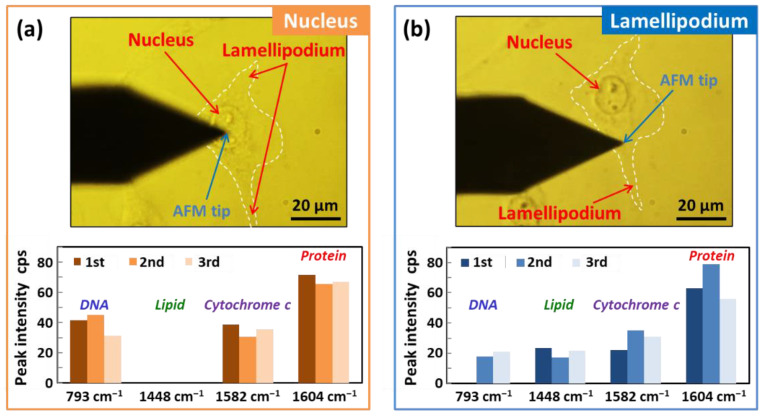
Intracellular TERS spectra comparison between the nucleus and lamellipodia (cytoplasm) of the same living HeLa cell. (**a**) Optical microscopy image showing the AFM tip aimed at the nucleus of the HeLa cell and a bar graph comparing representative Raman peak intensities for DNA (793 cm^−1^), lipids (1448 cm^−1^), cytochrome *c* (1582 cm^−1^), and proteins (1604 cm^−1^). (**b**) Optical microscopy image and a bar graph comparing the intensities of the same Raman peaks corresponding to the AFM tip aimed at the lamellipodia of the same cell. Note that the contours of the cells are denoted with white dotted lines in the images.

Figure 7 shows a quantitative comparison of the representative peak intensities in Raman spectra obtained from the nucleus and lamellipodia (cytoplasm) of the same living HeLa cell after the penetration of the cell membrane. The Raman measurements were repeated three times under the same conditions in exactly the same position in the cell in order to confirm reproducibility. The Raman spectra obtained are shown in Figure S4 in the Supplementary Materials. As shown in bar graphs comparing the peak intensities of the representative Raman peaks, there was no significant difference between the peak intensities for cytochrome *c* (1582 cm^−1^) obtained from the nucleus and lamellipodia. Similarly, a comparison of the intensities of the peak corresponding to proteins (1604 cm^−1^) also showed no significant difference. On the other hand, the peak for DNA (793 cm^−1^) appeared significantly stronger in the nucleus rather than in the lamellipodial region of the cell. This most likely reflects the fact that the cell nucleus contains a large quantity of DNA. In contrast, no peak for lipids (1448 cm^−1^) was observed in the cell nucleus, but it appeared in the lamellipodia. There are probably two main reasons for this. One is that the lamellipodia is a thin and flat cell membrane structure, which consists of a lipid bilayer. The other is that mitochondria contain high concentrations of phospholipids, which are located in the cytosol, but outside the nucleus [53]. The experimental results prove that the difference between the nucleus and lamellipodia can be clearly distinguished by AFM-based intracellular TERS imaging.

**Figure 8:**
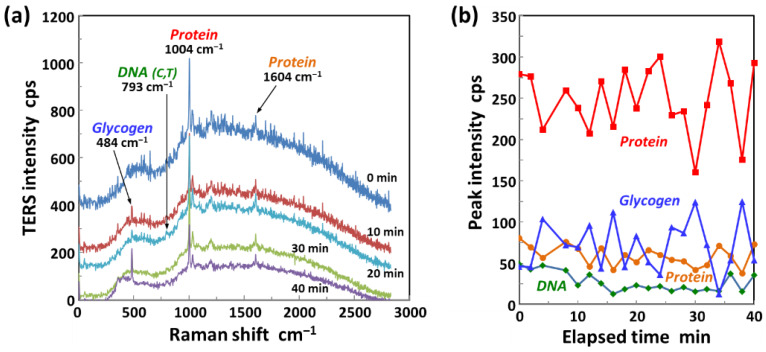
TERS imaging of molecular dynamics inside a living cell. (**a**) A series of TERS spectra obtained inside a HeLa cell at elapsed times ranging from 0 to 40 min after the penetration of its cell membrane (laser power: 50 mW, acquisition time: 10 s, cumulative number: 3). (**b**) Time-dependent changes in the intensities of the representative Raman peaks associated with glycogen (484 cm^−1^), DNA (793 cm^−1^) and proteins (1004 and 1604 cm^−1^).

Figure 8a shows a series of TERS spectra obtained inside a HeLa cell at elapsed times ranging from 0 to 40 min after the penetration of the cell membrane. According to the time-dependent changes in each peak intensity shown in Figure 8b, the peaks associated with DNA (793 cm^−1^) did not change significantly for at least 40 min after the AFM tip had penetrated the cell membrane. In contrast, Raman peaks associated with glycogen (484 cm^−1^) [56] and proteins (1004 cm^−1^) showed strong temporal fluctuations in peak intensity. This plot suggests that an increase in the peak intensity of proteins may cause a decrease in that of glycogen, and vice versa. Moreover, it should be noted that the increase or decrease in peak intensity for the other Raman signal assigned to proteins (1604 cm^−1^) followed the same temporal fluctuations of the 1004 cm^−1^ protein peak intensity. Although the meaning of the negative correlation between glycogen and proteins is still under consideration, the data nevertheless demonstrate that intracellular TERS imaging could allow us to quantitatively study dynamic processes inside living cells.

## 4. Conclusions and Future Research Directions

With the aim of adding new capabilities to AFM, we demonstrated the photocatalytic nanofabrication and intracellular TERS imaging of single living cells under near-physiological conditions by employing functionalized AFM probes. AFM probes functionalized with a thin layer of catalytic titanium dioxide (TiO_2_) showed the ability of direct photocatalytic cell membrane perforation when used on living cells under UV irradiation. Although it was a blunt-tipped AFM probe (tip radius: ~50 nm), it had a high probability of cell membrane perforation (more than 80%), which was significantly higher compared with the value (~46%) corresponding to an uncoated Si-based AFM probe with a sharp tip radius (~7 nm). Moreover, high cell viability (~100%) was achieved after the AFM tip had penetrated the cell membrane. It should also be noted here that the highly localized TiO_2_-mediated photocatalytic oxidation can be utilized for nanofabrication not only on living cells, but also on a wide variety of organic materials ranging from biomolecules (e.g., nucleic acids and proteins) to polymer-based engineering materials (e.g., self-assembled monolayers, polymer thin films, and engineering plastics). AFM probes functionalized with Ag nanoparticles were used to demonstrate the ability to visualize biomolecules inside living cells based on tip-enhanced Raman spectroscopy. This technique has the potential to provide local information from the inside of cells (e.g., the differences between the spectral signatures of the nucleus and cytoplasm) and physiological information on dynamic processes involving functioning biological molecules inside cells (e.g., the negative correlation between the amount of glycogen and proteins).

The next step in this study is to combine both of the capabilities described above, photocatalytic nanofabrication and intracellular TERS imaging of living cells, by using AgNP/TiO_2_-functionalized AFM probes. Such a strategy would allow us to ensure intracellular TERS imaging with a high success rate by combining it with high probability and little cellular damage photocatalytic cell membrane perforation. Further, it would enable in-process TERS imaging for the photocatalytic destructive oxidation process of living cells by monitoring structural changes in biomolecules (e.g., nucleic acids, proteins, lipids). Moreover, the excitation wavelengths for TiO_2_ can be expanded from the UV light region (<390 nm) to the visible light region (400–800 nm) by surface modification of TiO_2_ with Ag nanoparticles, owing to the surface plasmon resonance (SPR) effect of AgNPs [57]. Not having to rely on UV illumination would provide a significant advantage for photocatalytic nanofabrication in living cells; furthermore, it would reduce cell damage caused by UV irradiation. We also plan to apply this technique to destroy targeted membrane proteins selectively, thus enabling fundamental analysis of cellular functions and mechanisms in living systems. In this method, performed with ligand-functionalized AgNP/TiO_2_-AFM probes, the targeted single membrane protein (receptor) would be identified by monitoring the structural changes of ligand binding to specific receptors at the cell surface [41,42], that is, through a process known as molecular recognition in protein–ligand complexes.

## Figures and Tables

**Table 1:** Indentation distance and time (mean values and their standard deviations) during which force remained constant, and the probability that the flat portion of the force–distance curve appeared.

	Indentation Speed (nm/s)			
	50	100	150	300
Indentation distance (nm)	94 ± 30	80 ± 31	40 ± 26	10 ± 9
Indentation time (s)	1.88 ± 0.61	0.80 ± 0.31	0.27 ± 0.17	0.04 ± 0.03
Probability (%)	100	100	100	55
	(*n* = 20)	(*n* = 10)	(*n* = 7)	(*n* = 20)

**Table 2:** Effects of indentation speed on the probability of cell membrane perforation and the penetration force (mean values and their standard deviations).

	Indentation Speed (nm/s)			
	50	100	150	300
Probability (%)	100	100	70	80
	(*n* = 20)	(*n* = 10)	(*n* = 10)	(*n* = 25)
Penetration force (nN)	157 ± 58	177 ± 85	149 ± 64	128 ± 41
	(*n* = 20)	(*n* = 10)	(*n* = 7)	(*n* = 20)

## Supplementary Materials

The following are available online at https://www.mdpi.com/2072-666X/11/5/495/s1, Figure S1: Schematic diagram of a homemade Raman spectroscopy system; Figure S2: Typical force-distance curve obtained just before intracellular TERS imaging; Figure S3: Intracellular TERS spectra of living HeLa cells as a function of laser power; Figure S4: Intracellular TERS spectra obtained from the nucleus and lamellipodia of the same HeLa cell.
