# Supplementary material for: Photocatalytic Nanofabrication and Intracellular Raman Imaging of Living Cells with Functionalized AFM Probes
Source: Micromachines (Basel). 2020 May 13;11(5):495. doi: 10.3390/mi11050495 (PMC7281467; doi:10.3390/mi11050495)
Supplement: Supplementary file 1 [file micromachines-11-00495-s001.pdf]

micromachines

MDPI

Supplementary Materials

Photocatalytic Nanofabrication and Intracellular Imaging of Living Cells with Functionalized AFM Probes

Takayuki Shibata *, Hiromi Furukawa, Yasuharu Ito, Masahiro Nagahama, Terutake Hayashi, Miho Ishii-Teshima, and Moeto Nagai

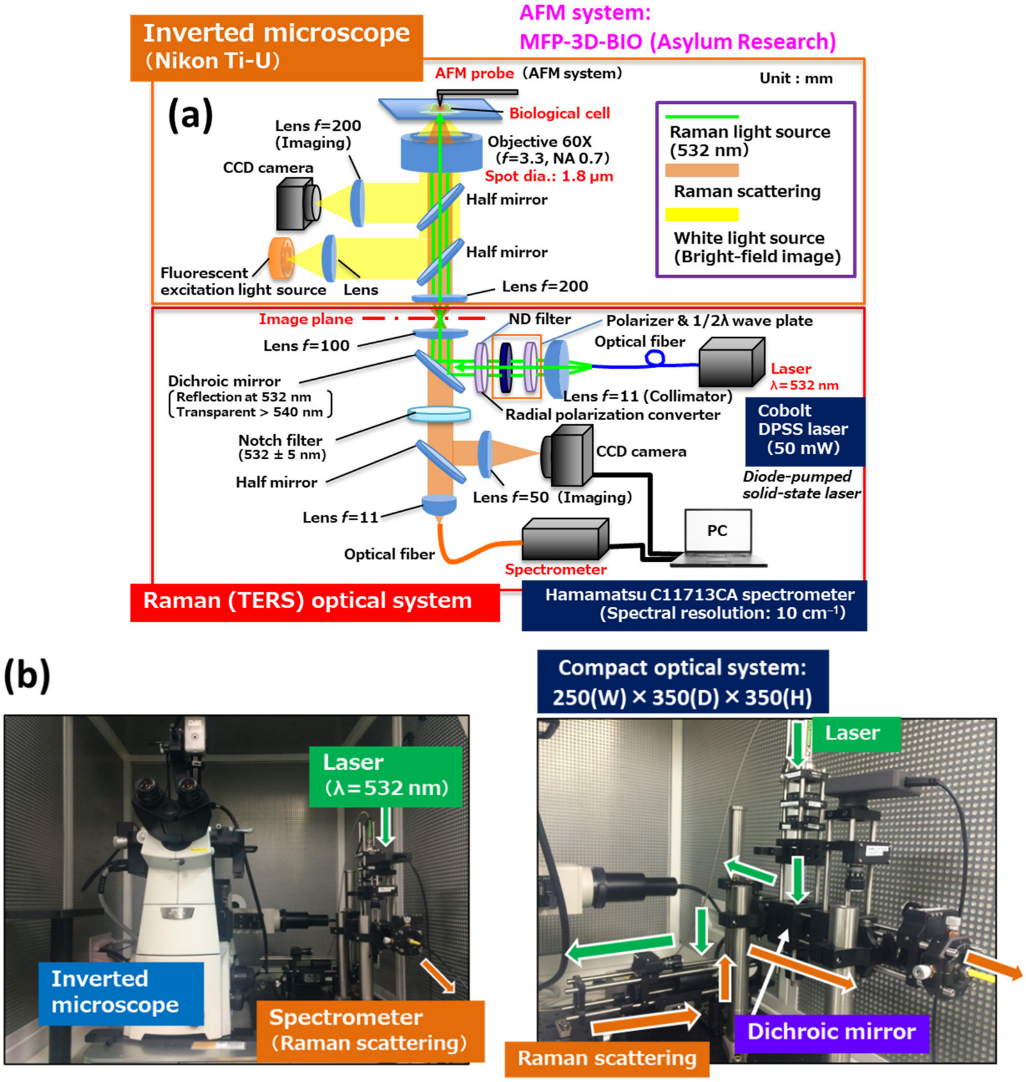

Figure S1. Homemade Raman spectroscopy system. (**a**) Schematic diagram of the optical configuration of the Raman spectroscopy system integrated with an inverted microscope for intracellular TERS imaging using AgNP-functionalized AFM probes. (**b**) Photographs of compact-size, home-built optical system for Raman spectroscopy (250 mm × 350 mm × 350 mm), which was enclosed inside an acoustic isolation enclosure for AFM measurements.

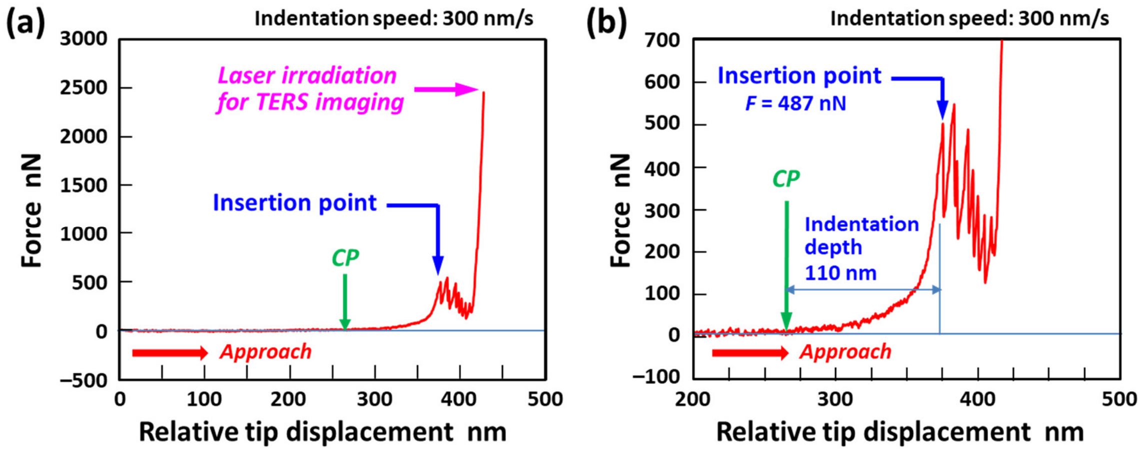

Figure S2. Typical force-distance curve obtained just before intracellular TERS imaging with an AgNP-functionalized AFM probe. (**a**) Overall features of the force curve when an AFM tip was inserted into a living HeLa cell with an indentation speed of 300 nm/s. (**b**) Close-up of the insertion point in the force curve showing a force drop observed when the indentation depth reached approximately 110 nm, measured from the contact point to the insertion point. The penetration force of the cell membrane was 487 nN. It should be noted that the vertical movement of the AFM probe was stopped once after the force reached around 2.5 μN, and then an excitation laser was irradiated on the AFM tip for TERS imaging. The symbol *CP* and the arrow in the graphs represent the contact point between the AFM tip and the cell surface.

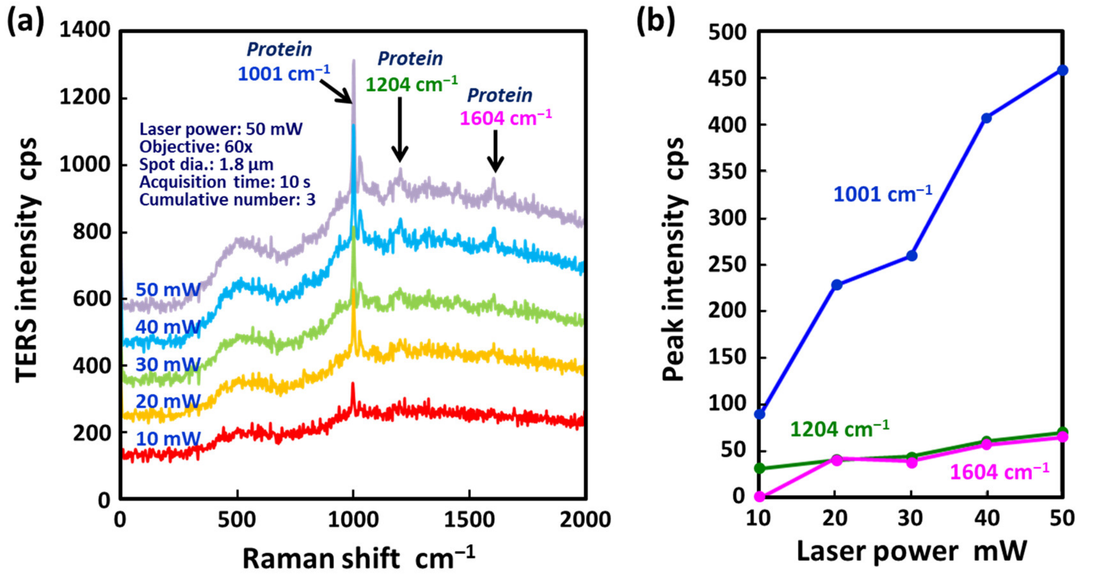

Figure S3. Effect of excitation laser power in Raman spectroscopy. (**a**) Intracellular TERS spectra of HeLa cells as a function of laser power, and (**b**) changes in the intensities of three representative Raman peaks that are associated with proteins (1001, 1204, and 1604 cm^{-1}).

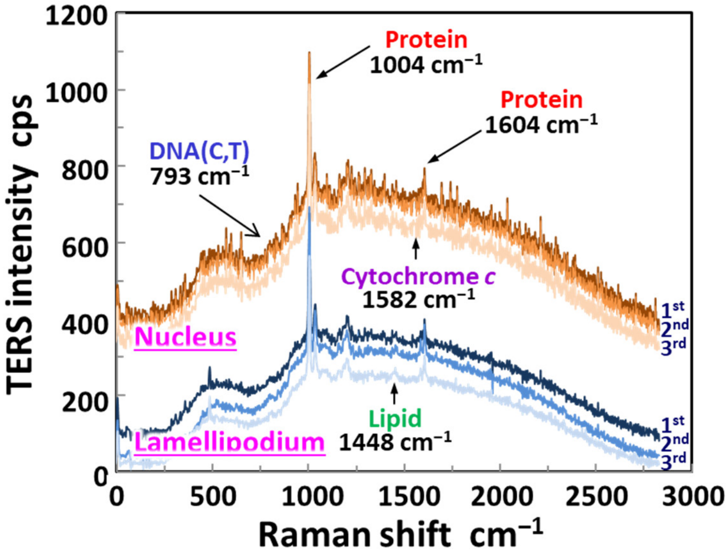

Figure S4. Intracellular TERS spectra obtained from the nucleus and lamellipodia of the same living HeLa cell after penetration of the cell membrane. The Raman measurements were repeated three times under the same conditions in exactly the same position in the cell (laser power: 50 mW, acquisition time: 10 s, cumulative number: 3).
